# *Raoultella ornithinolytica* Infection in the Pediatric Population: A Retrospective Study

**DOI:** 10.3389/fped.2020.00362

**Published:** 2020-07-10

**Authors:** Dan-dan Pi, Fang Zhou, Ke Bai, Chengjun Liu, Feng Xu, Jing Li

**Affiliations:** ^1^Intensive Care Unit, Children's Hospital of Chongqing Medical University, Chongqing, China; ^2^Ministry of Education Key Laboratory of Child Development and Disorders, National Clinical Research Center for Child Health and Disorders, China International Science and Technology Cooperation Base of Child Development and Critical Disorders, Chongqing Key Laboratory of Pediatrics, Children's Hospital of Chongqing Medical University, Chongqing, China

**Keywords:** *Raoultella orithinolytica*, infection, pediatric, clinical characteristics, antimicrobial susceptibility

## Abstract

*Raoultella ornithinolytica* is a pathogen causing an increasing number of pediatric infections. The objective of this study was to investigate the clinical characteristics of *R. ornithinolytica* infections in children. As a retrospective analysis, clinical features and drug susceptibility data of the five cases were analyzed and related literature was reviewed. A total of 14 cases (eight females, six males) were analyzed: nine cases were retrieved from PubMed, Web of Science, and three domestic databases; five cases occurred in our hospital. The primary diseases of the older children were mainly of neoplastic and immune origin, while cases of infants and young children were mostly complicated by congenital malformation. Fever was the main symptom, and neonatal infection was mainly manifested by dyspnea and hypoxemia, with multiple skin flushes, systemic erythema, and leukocytosis. Of the 14 cases, six were ventilator-assisted, five had indwelling urethral catheters, three had surgical treatment or chemotherapy, and one had multiple rounds of continuous renal replacement therapy (CRRT). Blood infection is the main route of *R. ornithinolytica* infection in children. Skin flushing and systemic erythema might be positive clues for newborn infection. Patients with multiple congenital abnormalities are susceptible to infection. Tumors, immune deficiency, and invasive operations increase the risk of infection. Blood culture was the main method of disease identification. Based on the drug susceptibility results, the preferred antibiotics are third generations of cephalosporins, carbapenems, quinolone, and aminoglycoside. Lastly, patients with sepsis mostly have poor prognosis.

## Introduction

The genus *Raoultella* is a member of the *Enterobacteriaceae* family. Three species belong to this genus: *Raoultella planticola, Raoultella ornithinolytica*, and *Raoultella terrigena*. *Raoultella ornithinolytica* is a gram-negative bacterium that lives in an aerophilic environment. *Raoultella* spp. is an opportunistic pathogen, common in cancer, immunocompromised patients, and most commonly in patients with biliary tract infection, pneumonia, and bacteremia (1–3). Formerly classified as *Klebsiella ornithinolytica*, the bacterium was renamed as *Raoultella* based on new genetic identification methods. In 2011, the genus *Raoultella* was identified based on the 16S rRNA and rpoB gene sequence analysis of *K. ornithinolytica, K. planticola*, and *K. terrigena* (4–6).

*Raoultella ornithinolytica* is reported to inhabit aquatic environments and can be found in hospital environments as well. In 2009, Morais and Vos et al. reported cases of human infections of *R. ornithinolytica* (7, 8). Most of the published case reports of this infection have focused on adult patients (9–14). *Raoultella ornithinolytica* infections in children have been reported for only a small number of cases. The clinical characteristics, drug sensitivity, and treatment outcomes of *R. ornithinolytica* infection in children have not previously been well-summarized and reported.

The purpose of our research was to describe the clinical characteristics, antimicrobial susceptibility, and prognoses of pediatric infections caused by *R. ornithinolytica*. The five cases reported herein are all from an 1800-bed children's specialist teaching hospital in China, representing the features of children with *R. ornithinolytica* infection in Southwest China. Secondly, by searching the main databases at home and abroad, we have made a summary of clinical characteristics of *R. ornithinolytica* infection in children reported all over the world. This work should aid the pediatricians in the diagnosis and therapy of *R. ornithinolytica* infection.

## Materials and Methods

We have conducted a retrospective study of culture records at the Children's Hospital of Chongqing Medical University (an 1800-bed teaching hospital in China) between January 1, 2013 and June 1, 2019. BACTEC 9120 system (BD, NJ, USA) has been used as a culture device in our hospital since 2013. Bacterial identification and antibiotic susceptibility testing were performed by the VITEK2 Compact (Bio Mérieux, France). BACTEC 9120 or the VITEK2 Compact systems were used for the cultures that were obtained for the records. In this study, only clinical symptoms of positive blood flow, urine, transbronchial catheter aspiration, bronchoalveolar lavage, pleural fluid, peritoneal fluid, bile, or deep tissue culture were studied. Positive cultures from sputum samples were not considered as respiratory tract infections. Cases were retrospectively analyzed to assess the clinical characteristics of each patient.

The study was conducted by searching the abroad databases (Pubmed database and Web of Science) and domestic databases (China zhiwang, Weipu database, and Wanfang database) with the key word “*Raoultella ornithinolytica*,” and June 1, 2019 was set as the cut-off date. A total of 25 related reports with eight references to nine pediatric cases were found (15–22). These nine cases were combined with the five cases from our hospital; the clinical features and identification methods were summarized in Tables 1, 2.

**Table 1:** Summary of *Raoultella ornithinolytica* bacteremia (according to age).

**Case**	**References**	**Sex**	**Age**	**Underlying diseases**	**Main symptoms and signs**	**WBC/×10^**9**^ L^**−1**^**	**Specimen**	**Antibiotics and duration (days)**	**Outcome**
1	(19)	M	18h	Data unavailable	Grunting, low activity, polypnea, diffuse red discoloration of skin	27	Blood	CTX, AMK (2d) → colistin (8d)	Survived
2	(20)	M	3d	Hyaline membrane disease, 33w premature	Dyspnea, hypoxemia	4.82	Blood	PIPC/TAZ, AMK (2d) → CPFX (8d)	Survived
3	(18)	F	14d	35w premature, oligohydramnios	Feeding intolerance, low activity, apnea, flushing, purpura	3.9	Blood	MEPM, VCM → MEPM, NTM	Died
4	(15)	M	15d	Visceral heterotaxy, asplenia, congenital cardiac anomaly	Fever, flushing	Data unavailable	Blood	CFPM, MNZ, AMK → MEPM, AMK (7d)	Survived
5	(Our case, 2017)	M	3m12d	Severe pneumonia, congenital heart disease	Pyrexia, polypnea, hypoxemia	39.6	Blood	CPFX (14d) → CPZ/SBT (14d)	Survived
6	(Our case, 2015),	F	6m14d	Severe pneumonia, 29^+2^w premature, BPD, ASD, PDA	Abdominal distension, cough	8.8	Bronchoalveolar lavage	CTM (12d)	Survived
7	(22)	F	8m	VUR	Fever, lack of feeding	18.5	Urine	CRO (3d) → CPOP (6d)	Survived
8	(21)	F	1y4m	unavailable	Persistent cough and fever, rash	Data unavailable	Broncho-alveolar lavage	AMPC/CVA	Survived
9	(16)	F	3y	IgA nephropathy	Pyrexia, proteinuria	8.5	Blood	CRO (14d)	Survived
10	(16)	F	7y	Acute myeloid leukemia	Pyrexia, polypnea	<0.1	Blood	MEPM (17d)	Survived
11	(17)	F	8y	Retinoblastoma, neurogenic bladder	Hyperpyrexia, frequent urinary tract infection	12.63	Blood (catheter-related)	AMK (6d)	Survived
12	(Our case, 2016)	F	11y	Juvenile idiopathic arthritis (generalized)	Cough, fever	12.3	Bronchoalveolar lavage	CZX (5d) → CAE (10d)	Survived
13	(Our case, 2017)	M	11y	Cerebellar hemangioblastoma	Polypnea, repeated fever	43.3	Blood	MEPM, VCM (7d) → AMK (21d)	Survived
14	(Our case, 2017)	M	16y	Evan's syndrome	Anemia, kidney failure, dyspnea, repeated fever	2.9	Blood	MEPM (3d) → CPFX (14d)	Survived

*Cases 5–6 and 12–14 were reported from our hospital and the other cases were found in the literature. The prognosis of the patients was excellent with antibiotic therapy*.

*WBC, White blood cell; CRO, ceftriaxone; MEPM, meropenem; AMK, amikacin; CFPM, cefepime; MNZ, metronidazole; VCM, vancomycin; NTM, netilmicin; CPFX, ciprofloxacin; CPZ/SBT, cefoperazone/sulbactam; CZX, cetizoxime; CAE, ceftazidime; CTM, cefotiam; CPOP, cefpodoxime proxetil; AMPC/CVA, amoxicillin/clavulanate; PIPC/TAZ, peperacillin/tazobatam*.

**Table 2:** Summary of the microbiological testing of the cases.

**Case**	**References**	**Culture device**	**Identification**	**Antimicrobial susceptibility**						
				**CFPM**	**MEPM**	**CSMZ**	**ABPC**	**CPFX**	**AMK**	**LVFX**
1	(15)	Unknown	Vitek 2 GN card	S	S	S	n.p.	S	S	S
2	(16)	Signal blood culture system	Microscan walkaway	n.p.	n.p.	S	R	R	n.p.	n.p.
3	(16)	Signal blood culture system	Microscan walkaway	n.p.	S	R	R	n.p.	n.p.	R
4	(17)	Signal blood culture system	VITEK2 compact	S	S	S	R	S	S	n.p.
5	(18)	Unknown	Unknown	S	S	S	n.p.	S	S	S
6	(19)	BacT/Alert	Microscan walkaway 40 SI	R	R	S	R	R	R	R
7	(20)	Bactec automated blood culture system	VITEK2 AST N255	R	R	R	R	S	R	n.p.
8	(21)	Unknown	Unknown	n.p.	n.p.	n.p.	n.p.	n.p.	n.p.	n.p.
9	(22)	Unknown	MicroScan dried gram-negative MIC/combo panels	S	n.p.	n.p.	n.p.	n.p.	n.p.	n.p.
10	(Our case, 2017)	BACTEC9120	VITEK2 compact	R	n.p.	R	R	S	S	S
11	(Our case, 2017)	BACTEC9120	VITEK2 compact	R	R	R	R	M	S	S
12	(Our case, 2017)	BACTEC9120	VITEK2 compact	R	R	R	R	S	S	S
13	(Our case, 2016)	BACTEC9120	VITEK2 compact	S	n.p.	R	R	S	S	S
14	(Our case, 2015)	BACTEC9120	VITEK2 compact	S	S	S	R	S	S	S

*The BACTEC system was mostly used for blood culture examination and the MicroScan Walkaway and VITEK2 Compact were mostly used for identification. The strains were resistant to ABPC but mostly susceptible to AMK, CPFX, and LVFX*.

*CFPM, cefepime; MEPM, meropenem; CSMZ, compound sulfamethoxazole; ABPC, ampicillin; CPFX, ciprofloxacin; AMK, amikacin; LVFX, levofloxacin; R, resistant; S, susceptible; M, mediate; n.p., not provided*.

Data analyses were performed using IBM SPSS Statistics software version 19.0 (IBM Corp., Armonk, NY, USA). Statistical significance was calculated using the Chi-square test or the Fisher's exact two-tailed test. A *p* < 0.05 was considered statistically significant.

## Results

A total of 14 *R. ornithinolytica* infection cases in children were reported during the period between January 1, 2013 and June 1, 2019. As shown in Table 1, out of the 14 cases of children infected with *R. ornithinolytica*, eight cases were female and six were male; the oldest child was 16 years old and the youngest was a 18 h newborn. The geographical distribution included one case in the United States (15), two cases in Japan (16), one case in Poland (17), one case in India (19), one case in South Africa (20), one case in Italy (22), two cases in Turkey (18, 21), and five cases in China (our report). About half of the children infected with the bacteria were over 3 years old and adolescents (42.8%, 6/14). The primary diseases were mostly related to tumors and immune system dysfunction. There were four neonatal cases (28.6%, 4/14), which mainly had congenital malformations. Ten cases (71%) had fever as a symptom, and a significant increase in white blood cell count (6/14). The onset of neonatal disease was mainly characterized by dyspnea, red skin, and erythema.

Table 2 summarizes the identification and antibiotic susceptibility of *R. ornithinolytica*. Ordinary blood culture medium and BACTEC 9120 were the most commonly used culture devices, while the Microscan Walkaway and VITEK2 Compact analysis systems were mostly used for bacteria identification. Ten blood samples, three bronchoalveolar lavage fluid samples, and one urine sample were analyzed. The most frequently used antibiotics were cefepime, meropenem, ciprofloxacin, and amikacin.

Statistical susceptibility results showed that 10/14 (77%) cases were resistant to ampicillin, and the remaining four cases did not meet the drug susceptibility criteria. Five out of 14 (36%) patients were sensitive to cefepime and meropenem, 6/14 (43%) were sensitive to sulfamethoxazole, 7/14 (50%) were sensitive to levofloxacin, while 8/14 (57%) were sensitive to ciprofloxacin and amikacin. The courses of antibiotic treatment ranged from 6 days to 3 weeks. There was one neonatal death, while all other patients improved, which suggested a good overall prognosis.

## Discussion

*Raoultella ornithinolytica* was previously considered to be a rare opportunistic pathogen, which has not attracted enough attention from pediatricians. Currently, there are only few reports on children infected with *R. ornithinolytica* all over the world (15–22), and none of the articles have ever provided a summary of the clinical characteristics of children infected with *R. ornithinolytica*. In our study, we reported five cases from an 1800-bed teaching hospital in southwest China, which is the largest number of children identified with *R. ornithinolytica* to date. For the first time, together with the nine cases of *R. ornithinolytica* reported by others, we have summarized the clinical characteristics of the 14 cases infected with the bacteria in children. Unlike adults, children are mainly infected with *R. ornithinolytica* through the blood stream rather than biliary, respiratory, or urinary tracts (11, 23). Secondly, most of the newborns infected with *R. ornithinolytica* also develop skin flushing and systemic erythema, mainly due to the presence of histamine-like substances in *R. ornithinolytica* (24–26). Thus, this could also be a diagnostic clue of the infection in newborns.

We found that *R. ornithinolytica* infection was more common in children above 3 years old and newborns than other ages. There was no obvious difference in gender distribution. The primary diseases of older children were mainly tumor and immune system related, while the newborns mostly had congenital deficiencies. These features are consistent with the findings of Senget al. (23); in this report, about a half of the 112 patients diagnosed with *R. ornithinolytica* infection in four hospitals over 11 years had tumor and immune related diseases. Immune dysfunction may be a high-risk factor for the infection of *R. ornithinolytica*. There were two cases of abnormal immune function in our hospital and one case reported in the literatures (16). Infants and children under 3 years old are more likely to have congenital malformations [two cases in our hospital, and four cases in the literature reports (15, 18, 20, 22)]. Adults are more likely to have urinary tract infections; possibly because urinary catheters are more commonly used in adult patients. It has been reported before that *R. ornithinolytica* has the ability to form biofilms inside the catheters (27). The ability to colonize the inner surfaces of the indwelling urinary catheters is a main reason of hospital-acquired urinary tract infections (23). Case analysis on adults suggests that invasive procedures may increase the risk of contracting *R. ornithinolytica*. This suggestion is in accordance with our findings analyzing the infection cases in children: 6/14 patients underwent mechanical ventilation, 5/14 had indwelling catheters, 3/14 underwent surgery, 3/14 underwent tumor chemotherapy, and 1/14 underwent blood purification for four times. *Raoultella ornithinolytica* bacteria form biofilms on invasive devices such as venous catheters and mechanical ventilations, thus increasing the risk of infection. This could also explain a higher number of *R. ornithinolytica* blood stream infections in children, as venous catheters are often used for their treatment. Further, fever is the most common clinical manifestation, however newborns with infection of this bacteria were mainly diagnosed with dyspnea and hypoxemia.

As an opportunistic pathogenic bacterium, *R. ornithinolytica* is widely distributed in the natural world. It is unknown whether it has regional distribution characteristics. Adult infection distribution shows, that 9/11 *R. ornithinolytica* bacteremia cases have been reported in Japan (11). *Raoultella ornithinolytica* infections in children worldwide are reported as separate cases in scientific literature. Of the 14 cases reported to date, ten came from Asia—five from China, two from Japan, two from Turkey, and one from India. It is not clear whether the Asian population is particularly vulnerable to *R. ornithinolytica*. A deep investigation and a larger sample size are required to reliably evaluate the clinical outcome of *R. ornithinolytica* bacteremia.

Accurate bacterial identification is essential to detect higher numbers of bacterial infection cases. It is difficult to distinguish *R. ornithinolytica* from *K. oxytoca* in clinical laboratories using conventional methods of phenotypic identification (6). Our hospital had no positive test results of *R. ornithinolytica* infections until 2015, as a new bacterial identification equipment VITEK2 Compact was introduced only in 2013. VITEK2 system has five biochemical tests that can identify ornithine decarboxylase (ODC)-negative *R. ornithinolytica* isolates. Nevertheless, this method had to be validated by molecular identification (16S rRNA gene sequencing) due to the lack of specificity (23). The identification of this bacterium in our hospital was only based on the biochemical characteristics of the pathogen using VITEK 2 system, therefore false identification might have occurred. Previously, some cases of *R. ornithinolytica* being misclassified as *K. oxytoca* have been reported, however, *K. oxytoca* misclassification as *R. ornithinolytica* has never occurred (28). Thus, we believe that the identification of *R. ornithinolytica* was accurate. In recent years, matrix-assisted laser desorption/ionization-time of flight mass spectrometry (MALDI-TOF MS) has been used to detect *R. ornithinolytica* infection (23, 29).

*Raoultella ornithinolytica* has natural resistance to ampicillin due to the production of β-lactamase. According to the analysis of the drug susceptibility results in 14 children, combined with related literature (30, 31), it is considered that cephalosporin, carbapenems, quinolones and aminoglycosides can be chosen for infection treatment (4). Patients have a satisfying prognosis with timely antibiotic administration, however a death rate of 9% was reported (23). In our summary, 1/14 (7.1%) patients died; the patient was a preterm newborn at gestational age of 35 weeks. In this case, obstetric ultrasonography showed restricted intrauterine growth before birth, and limited eating and movements after birth along with hypoxemia, violent purpura that were gradually spreading from the limbs to the whole body, disorders of coagulation, late pulmonary hemorrhage complications with multi-functional organ failure, which led to patients death 19 days after birth. Looking back at the current literature reports, one adult death has been reported (32). The patient was 37 years old and had acute lymphoblastic leukemia. He was treated with bone marrow stem cell transplantation one year before hospitalization, and then was infected with *R. ornithinolytica* during hospitalization. The main symptom was stubborn high fever. The patient was treated with a variety of antibiotics (imipenem, amoxicillin clavulanate potassium, and ciprofloxacin, etc.) but the infection was poorly controlled. The patient died 1 week after being transferred to the intensive care unit.

Previously known as a cause of severe community infections, currently *R. ornithinolytica* is known to cause hospital-acquired infections, especially in patients who have received invasive procedures (23). In this paper we have made a summary of the clinical characteristics of *R. ornithinolytica* bacteremia in children. The number of reported *R. ornithinolytica* infections was probably lower than the actual ones due to limited detection methods. The identification of *R. ornithinolytica* isolates may be improved with the introduction of a very promising technique–MALDI-TOF MS. Proper handling of *R. ornithinolytica* spread is very important, as severe complications after infection can lead to sepsis and patients death due to multiple organs failure.

## Conclusion

*Raoultella ornithinolytica* infection, despite being rare, is still a life-threatening childhood disease that results in significant morbidity and mortality in neonates and immunosuppressed children.

## Limitations

This is a retrospective case study conducted using different sources. Most cases are reported from the non-western part of the world. Further prospective studies are needed to confirm the findings.

## Data Availability Statement

All datasets presented in this study are included in the article/supplementary files.

## Ethics Statement

This study was approved by the Institutional Review Board of the Children's Hospital of Chongqing Medical University (Chongqing, China, No. 202016). Written informed consent to participate in this study was provided by the participants' legal guardian/next of kin. Written informed consent was obtained from the minor(s)' legal guardian/next of kin for the publication of any potentially identifiable images or data included in this article.

## Author Contributions

DP and FZ contributed to the conception and design of this study. DP and CL organized the data and performed the statistical analysis. FZ and KB drafted the manuscript. FX and JL wrote sections of the manuscript. All authors contributed to manuscript revision, read, and approved the submitted version.

## Conflict of Interest

The authors declare that the research was conducted in the absence of any commercial or financial relationships that could be construed as a potential conflict of interest.
